# Identification and management of prediabetes: results of the Latin America Strategic Prediabetes Meeting

**DOI:** 10.26633/RPSP.2017.172

**Published:** 2017-12-12

**Authors:** Patricio López-Jaramillo, Ramfis E. Nieto-Martínez, Gestne Aure-Fariñez, Carlos O. Mendivil, Rodolfo A. Lahsen, Ruy L. Silva-Filho, Luiz A. Andreotti, Mónica E. Manrique, Miguel A. Pasquel-Andrade, Ignacio Rangel, Maricela Vidrio, Rutila Castañeda, Manuela Restrepo, Miguel E. Pinto

**Affiliations:** 1 Metabolic Syndrome and Diabetes Clinic Fundación Oftalmológica de Santander Bucaramanga Colombia Metabolic Syndrome and Diabetes Clinic, Fundación Oftalmológica de Santander, Bucaramanga, Santander, Colombia.; 2 Human Physiology Universidad Centro-Occidental Lisandro Alvarado Barquisimeto Venezuela Human Physiology, Universidad Centro-Occidental Lisandro Alvarado, Barquisimeto, Venezuela.; 3 Centro Medico Docente la Trinidad Centro Medico Docente la Trinidad Caracas Venezuela Centro Medico Docente la Trinidad, Caracas, Venezuela.; 4 Universidad de los Andes Universidad de los Andes Bogotá Colombia Universidad de los Andes, Bogotá, Colombia.; 5 Clínica Las Condes Clínica Las Condes Santiago Chile Clínica Las Condes, Santiago, Chile.; 6 Federal University of Pernambuco Federal University of Pernambuco Recife Brazil Federal University of Pernambuco, Recife, Brazil.; 7 Diabetes Unit Hospital das Clínicas da Faculdade de Medicina da Universidade de São Paulo São Paulo Brazil Diabetes Unit, Hospital das Clínicas da Faculdade de Medicina da Universidade de São Paulo, São Paulo, Brazil.; 8 Nutrition and Diabetes Unit Clínica Alemana Santiago Chile Nutrition and Diabetes Unit, Clínica Alemana, Santiago, Chile.; 9 Instituto VIDA Instituto VIDA Quito Ecuador Instituto VIDA, Quito, Ecuador.; 10 Medical School Monterrey Technological Institute Monterrey Mexico Medical School, Monterrey Technological Institute, Monterrey, Mexico.; 11 Research Cardiometabolic Unit Occidente SC Guadalajara Mexico Research Cardiometabolic Unit, Occidente SC, Guadalajara, Mexico.; 12 Center for Clinical Research Center for Clinical Research Mexico City Mexico Center for Clinical Research, Mexico City, Mexico.; 13 Merck Colombia Merck Colombia Bogotá Colombia Merck Colombia, Bogotá, Colombia.; 14 School of Medicine Alberto Hurtado Cayetano Heredia University Lima Perú School of Medicine Alberto Hurtado, Cayetano Heredia University, Lima, Perú.

**Keywords:** Prediabetic state, diabetes mellitus, type 2, diabetes, prevention & control, Latin America, Estado prediabético, diabetes mellitus tipo 2, diabetes, prevención & control, América Latina, Estado pré-diabético, diabetes mellitus tipo 2, diabetes, prevenção & controle, América Latina

## Abstract

*To understand the status of prediabetes diagnosis and treatment in Latin America and to evaluate the use of metformin for diabetes prevention in this context*.

*A panel of 15 diabetes experts from seven countries in Latin America met on 14 – 15 August 2014 in Lima, Peru, to review the available literature, discuss the role of prediabetes in type 2 diabetes mellitus and cardiovascular disease, analyze collected information, and make conclusions for prediabetes diagnosis and treatment in Latin America*.

*Prediabetes diagnosis, screening, and treatment, including lifestyle changes, pharmacological treatment, and cost-effectiveness were discussed. Five resulting statements were issued for Latin America: prediabetes is a clinical and public health problem; health care systems do not currently diagnose/treat prediabetes; use of prediabetes risk detection tools are needed region-wide; treatment includes lifestyle changes, multidisciplinary education, and metformin; and registries of patient records and further studies should be supported*.

*The expert panel concluded that in Latin America, preventive treatment through lifestyle changes and metformin are cost-effective interventions. It is important to improve prediabetes identification and management at the primary care level*.

## RESUMEN

Comprender el estado del diagnóstico y el tratamiento de la prediabetes en América Latina y evaluar el uso de la metformina para la prevención de la diabetes en este contexto.

Un panel de 15 expertos en diabetes de siete países de América Latina se reunió del 14 al 15 de agosto de 2014 en Lima, Perú, para revisar la literatura disponible, discutir el papel de la prediabetes en la diabetes mellitus tipo 2 y la enfermedad cardiovascular, analizar la información recolectada y formular conclusiones para el diagnóstico y el tratamiento de la prediabetes en América Latina.

Se analizaron el diagnóstico, el tamizaje y el tratamiento de la prediabetes, inclusive los cambios en el estilo de vida, el tratamiento farmacológico y la relación costo-eficacia. Se emitieron cinco conclusiones para América Latina: la prediabetes es un problema clínico y de salud pública; los sistemas de atención de la salud actualmente no diagnostican o no tratan la prediabetes; el uso de herramientas de detección del riesgo de prediabetes es necesario en toda la región; el tratamiento incluye cambios en el estilo de vida, educación multidisciplinaria y metformina; y se debe brindar apoyo para llevar registros de historias clínicas y realizar estudios adicionales.

El panel de expertos concluyó que en América Latina el tratamiento preventivo basado en cambios en el estilo de vida y administración de metformina son intervenciones eficaces en relación al costo. Es importante mejorar la identificación y el manejo de la prediabetes en el nivel de atención primaria.

## RESUMO

Entender o estado do diagnóstico e tratamento do prediabetes na América Latina e avaliar o uso de metformina para prevenção de diabetes neste contexto.

Um painel de 15 especialistas em diabetes de sete países da América Latina reuniu-se de 14 a 15 de agosto de 2014 em Lima, Peru, para analisar a literatura disponível, discutir o papel do prediabetes em diabetes mellitus tipo 2 e doenças cardiovasculares, analisar informações coletadas e fazer conclusões para o diagnóstico e tratamento do prediabetes na América Latina.

O diagnóstico, rastreio e tratamento pré-diabetes, incluindo mudanças de estilo de vida, tratamento farmacológico e custo-efetividade foram discutidos. Foram emitidas cinco conclusões resultantes para a América Latina: o prediabetes é um problema clínico e de saúde pública; os sistemas de saúde atualmente não diagnosticam/tratam prediabetes; o uso de ferramentas de detecção de risco de prediabetes é necessário em toda a região; o tratamento inclui mudanças de estilo de vida, educação multidisciplinar e metformina; e devem ser suportados registros de pacientes e outros estudos.

O painel de especialistas concluiu que na América Latina, o tratamento preventivo através de mudanças de estilo de vida e metformina são intervenções efetivas em relação ao custo. É importante melhorar a identificação e gestão do prediabetes no nível de atenção primária.

In Central and South America, more than one-half of individuals with type 2 diabetes mellitus (DM2) remain undiagnosed (1). Prior to DM2 onset, there is an insulin-resistance stage followed by an increase in blood glucose levels when “prediabetes” may develop, especially in people suffering visceral obesity. Several studies in different populations have suggested that prediabetes increases the risk for developing DM2 and subsequent cardiovascular diseases (CVD; 2 – 5); and that DM2 can be prevented or delayed by implementing certain lifestyle changes (LSC), as well as with medication (6 – 9). Identifying and treating people at risk of developing DM2 and subsequent CVD complications is a public health challenge.

Taking into account the implications of prediabetes as a risk for DM2 and CVD—both of which have seen rising prevalence rates throughout Latin America—and acknowledging that early diabetes management can prevent these subsequent conditions (10, 11), an expert panel of 14 diabetes physicians was convened. The group met in Lima, Peru, representing seven countries: Brazil, Chile, Colombia, Ecuador, Mexico, Peru, and Venezuela. The expert panel reviewed the existing literature and discussed professional opinions and experiences to reach decisions regarding prediabetes diagnosis and management in Latin America.

The main objective of the expert panel was to engage key opinion leaders and to analyze the available information in order to form a comprehensive understanding of the Latin American perspective on prediabetes diagnosis and treatment, and specifically, to evaluate the potential for using metformin in diabetes prevention.

## MATERIALS AND METHODS

### Panel selection

Prospective panel members were chosen from a group of specialists in each country (*n* = 14) according to their recognized, scientific expertise in diabetes research, practice, and quality measures. Other factors influencing selection were the desire for a broad range of medical specialties and different practice settings (e.g., primary health care, ambulatory care) to be represented on the panel. In addition, each candidate’s scientific contributions to the topic were reviewed, as well as their status among national scientific societies.

To ensure that potential conflicts of interest were disclosed and addressed appropriately, panelists presented any relevant information at the start of the meeting. Potential conflicts are listed in the appropriate section at the end of this article.

### Development process

The panel convened for a 2 days in Lima, Peru, to share professional opinions and experiences. All sessions were video recorded and a transcript was developed afterwards for each session. Panel discussions were used to define terms and to address questions regarding prediabetes screening, current treatment, and diagnostic criteria.

At the beginning of each session, a leading expert in each topic presented and summarized the current scientific evidence and its quality for the panel members. Afterwards, the participants were divided into three groups to discuss the presented evidence, analyze the quality of the evidence, and if necessary, to add additional evidence from existing and grey literature (unpublished work or studies pending publication). Each group then presented its conclusions to the entire panel. The exchanges that followed were documented and the basic points were summarized.

The expert meeting followed a topic review methodology, assessing needs in three concatenated phases (Table 1). In the first phase, epidemiological data on diagnostic criteria (Box 1), prevalence, screening, and treatment of prediabetes in Latin America were addressed. In the second phase, the goals, hurdles, and actions needed to improve prediabetes detection and treatment were identified. In this phase, each topic was addressed from three perspectives: the patient as an individual (individual perspective); the health care providers (institutional perspective); and the health policymakers (health system perspective). Finally, in the third phase of the plenary session, recommendations for improving prediabetes diagnosis and treatment in Latin America were discussed, agreed upon, and summarized.

**TABLE 1.** Methodology of the expert panel on prediabetes, Lima, Peru, 2014

Phase	Contents	Methods	Stages/topics
1	Introduction to the subject and contextualization (high-level insight)	Conferences and questions/answers	– Presentations on the current state of prediabetes in Latin America – Discussion of prediabetes diagnostics and treatment
2	Analysis of the situation and identification of goals, hurdles, and actions	Discussion workshops. Two 30 – 45-minute sessions for each of the three groups	– How to improve awareness on prediabetes in Latin America? – How to improve prediabetes diagnosis by primary care physicians?
3	Reaching consensus on priority action items	Plenary session	– Discussion and establishment of basic points to be considered by a program to improve prediabetes detection and treatment in Latin America

***Source:*** Prepared by authors from the study data.

BOX 1. Diagnostic criteria for prediabetes,^a^ 2014

Fasting plasma glucose: 100 mg/dl (5.6 mmol/L) to 125 mg/dl (6.9 mmol/L) (Impaired Fasting Glucose)

or

Glucose 2 hours after a 75 g overload of anhydrous glucose: 140 mg/dl (7.8 mmol/L) to 199 mg/dl (11 mmol/L) (Impaired Glucose Tolerance)

or

HbA1c: 5.7% – 6.4%

a There is a continuous risk for prediabetes, extending below the lower limit and becoming disproportionately higher at the upper limit of the range.

***Source:*** Prepared by authors based on a review of the literature.

### Disagreement resolution

A consensus process was used to resolve disagreement on qualifying the scientific evidence. Additional literature was obtained and included, as needed. When group consensus was not clear, the full panel reviewed the evidence and worked through any differences until a consensus was reached.

## RESULTS

Two of the principal topics discussed by the expert panel were the use of the Finnish Type 2 Diabetes Risk Score (FINDRISC) scale in Latin America and prediabetes interventions, particularly LSC and the use of medications.

### Prediabetes and diabetes risk assessment

Diabetes risk scores are useful for screening individuals who have an increased risk of DM2 and might benefit from additional, invasive diagnostic tests. The FINDRISC scale, the most used and validated worldwide, is one of the most effective tools for assessing individual diabetes risk (12). Since abdominal obesity was originally categorized using the cutoffs for European populations, this tool must first be validated according to the cutoffs of the population in which it is applied. Considering that the cutoff for abdominal obesity in Latin America (using a visceral adiposity area ≥ 100cm^2^) was less than that of Europe (≥ 94 cm in men; ≥ 80 cm in women), the FINDRISC was modified using this value.

A study to validate a low-cost tool for identifying diabetic patients in rural areas of Honduras (13) had very good test characteristics (area under the receiver operating characteristic [ROC] curve = 0.89). Using the screening criterion of 0.42, their risk equation had a sensitivity of 74.1% and specificity of 97.2%. Moreover, a study to validate a risk tool for detecting the diabetes incidence in Mexico (14) revealed an area under the receptor-operator curve of 0.9 (92% sensitivity; 71% specificity).

The Latin American FINDRISC (LA-FINDRISC) was validated in Bogotá and Bucaramanga, Colombia (15, 16), and Barquisimeto, Venezuela (17). The LA-FINDRISC presented a good discriminative power identifying subjects with glucose regulation risk (impaired fasting glucose [IFG] , impaired glucose tolerance [IGT], unknown DM2) in both places, with an area under the ROC curve ≥ 0.77, and an efficacy similar to that of the original version, even better for women. A value greater than 12 points showed the best sensitivity/specificity in both genders for detecting a high risk of impaired glucose regulation. These individuals would be eligible for the oral glucose tolerance test (OGTT) and/or HbA1c. Validation of a given risk score prior to applying it to a specific population is recommended to ensure sensitivity and specificity since the weight of different score components may vary among populations (18). The Colombian Diabetes Risk Score (ColDRISC) study validated and weighted independent elements of the FINDRISC among 3 000 people in northern Colombia (19). The study revealed that the performance of all three risk scores (ColDRISC, FINDRISC, and LA-FINDRISC) in screening for DM2 in Colombia was good. Furthermore, there are several other Latin American groups currently working on FINDRISC validation and/or application (19 – 21). However, these risk scores are not yet systematically applied in Latin America.

### Prediabetes interventions and challenges

The expert panel agreed that prediabetes interventions to prevent DM2 and its CVD complications in Latin America must include:

- Education about healthy lifestyle (beginning at school age) and training of educators on risk factors for DM2 and CVD, specifically targeting the most susceptible populations.
- Investments to create more favorable and safe environments for physical activities (e.g., parks, bike trails, and sports facilities).
- Recognition by every community sector that abdominal obesity, overweight, obesity, and prediabetes are conditions that increase the risk for DM2 and CVD, and that managing these will prevent the leading cause of morbidity and mortality.
- Evaluation of incentive programs that compensate health care providers and physicians that get good results from implementing prevention measurements.
- Financial support for studies that validate FINDRISC as a DM2 risk-detection tool and evaluate metformin for diabetes prevention.

The expert panel also identified some of the most frequent conditions that preclude proper identification of individuals with prediabetes in Latin America:

- Unavailability of anhydrous glucose for the glucose tolerance test.
- Suitability and technical uniformity on HbA1c measurement.
- Lack of economic investment in implementing screening activities to identify prediabetes in the primary health care system.
- Lack of transportation to clinics where diagnostic tests are performed.
- Lack of mobile clinics to reach areas that are difficult to access.

### Lifestyle changes for diabetes prevention

During recent years, there has been a rapid increase in the prevalence of DM2 in Latin America, closely associated with an increased prevalence of obesity (22). The rise of obesity is mainly attributed to increased caloric intake and sedentary lifestyles (22). The effects of weight reduction from LSC on the risk for developing DM2 in individuals with prediabetes has been evaluated by several studies and has shown encouraging results (6). However, there are no published studies on the effects of lifestyle interventions on DM2 risk in Latin America.

According to the Diabetes Prevention Program (DPP), the main DM2 prevention predictor for those with prediabetes is weight reduction—the risk of diabetes decreases by 16% with every kilogram of lost weight (6). However, weight reductions seen in the first months during clinical trials for DM2 prevention thin out as follow-up time increases (8, 9). Community-implemented programs have shown less weight loss than that reported by clinical trials, so it is possible that figures on DM2 prevention from the clinical trials are not reproducible in the real world (6). Moreover, it is possible that the reduced, beneficial effect on slowing the progression to DM2 seen in several studies—i.e., the Da Qing (from 51% to 43% in a 20 year follow-up; 7), the DPS (from 58% to 43% in a 7-year follow-up; 8), and the DPP (from 58% to 34% in a 10-year follow-up; 9)—could be due to the progressive weight gain at the follow-up, after the active intervention. However, these observational studies did show that long-term lifestyle changes are effective in DM2 prevention.

The consensus on DM2 prevention published by the International Diabetes Federation (18) proposes identification, risk assessment, and intervention to reduce the risk of prediabetes. It also recommends 30 minutes of moderate exercise daily, maintaining a healthy weight, decreasing weight in overweight and obese individuals by 5% – 10%, and maintaining a normal weight/height ratio in children (18). Similar recommendations have been proposed in Latin America (23).

In conclusion, analysis of the evidence shows that lifestyle changes are an effective tool to prevent DM2 in individuals with prediabetes and should always be implemented. However, their efficacy decreases over time and in real life, the percentage of patients getting to and maintaining the weight loss and the healthy habits is very low, so pharmacological measures may be necessary in a very high percentage.

### Pharmacological treatment

Metformin is a biguanide that reduces the production of glucose by the liver and causes moderate weight loss in overweight and obese patients (6). Using an 850 mg dose twice daily was shown to reduce the progression from prediabetes to diabetes (6) in 31% of participants versus the placebo group. The study was conducted in the United States where metformin was shown to be particularly effective in young adults, in those with a BMI higher than 35, and in women with a history of gestational diabetes. The beneficial effect of metformin was reduced by 25% after a short period of discontinuation from the drug. In the Indian Diabetes Prevention Program (IDPP; 24), the progression rate from prediabetes to diabetes in the placebo group was 18.3% per year. Metformin at lower doses (250 mg) effectively reduced the progression rate to DM2, by an absolute reduction of 14.5%. This was a two-fold reduction versus that of the DPP (7.2% decrease in new cases of DM2), with a higher dose of metformin (1700 mg/day). This difference translates into a lower necessary number needed to prevent a new case of DM2, namely 6.9 in IDPP and 13.9 in DPP. Overall, metformin was well tolerated in the DPP study, although 29% of patients did not reach the goal of taking over 80% of the prescribed drug during the study period.

To explain these results, in addition to the differences in trial methodology, it has been proposed that there are regional differences regarding sensitivity for developing insulin resistance, low grade inflammation, and DM2 at lower levels of visceral adiposity related to epigenetic changes and fetal programming (25). Thus, it is essential to conduct clinical trials to evaluate the effects of interventions in the Latin American context (26, 27). Studies like the DPP and the IDPP are not available for Latin America. Despite this gap, it seems prudent to identify individuals with abdominal obesity and prediabetes early in order to implement lifestyle changes and prescribe 500 – 1 700 mg of metformin daily, especially if blood glucose levels remain high after lifestyle changes.

### Cost-effectiveness of prediabetes treatment

Although economic data on DM2 prevention from the United States and other developed countries cannot be extrapolated to Latin America, they are useful as discussion guides. Thus, a study comparing the costs of health care in patients with IFG, IGT, or both, compared to patients with normal glucose levels, showed an increase in subjects with prediabetes (28). In the United States in 2007, the annual health care cost for an individual with diabetes was estimated at US$ 11 700, compared to US$ 2 900 for a non-diabetic (28, 29). In addition, drug expenses for complications related to DM2 treatment and specialists’ fees increased medical expenses by 360%. If renal insufficiency requires dialysis, the costs then increase by up to 771%. In the United States, 2006, the yearly health care cost for an individual with IGT was modeled and calculated to be US$ 1 400; costs increased to US$ 1 900 for non-complicated DM2 treated with monotherapy; to US$ 2 200 if microalbuminuria occurs; to US$ 2 700 if hypertension additionally occurs; and finally, to US$ 4 600 with additional angina (30).

Thus, there are financial reasons as well as health reasons to identify individuals with prediabetes and to follow up with lifestyle interventions and/or metformin. The cost-effectiveness analyses of several DM2 clinical trials comparing LSC to pharmacotherapy have reported the latter, mainly metformin, to be superior. For instance, the cost of LSC intervention in DPP in 2002 was US$ 1 400/person during the first year and decreased to US$ 700 per person in the following years (31). Metformin cost for the dose used in DPP (1700mg/day) was approximately US$ 300 per person annually. As discussed by Herman and colleagues (31), the cost of metformin was significantly lower than the acarbose cost in the “Study to Prevent Non-Insulin-Dependent Diabetes Mellitus trial” (US$ 1 400 per person annually), and “Rosiglitazone in the Diabetes Reduction Assessment with ramipril and rosiglitazone medication trial” (US$ 2 000 per person annually) in 2006 (28).

Table 2 shows the financial analysis performed at the institution that implements preventive treatment in subjects at cardiometabolic risk conditions in Bucaramanga, Colombia (*Clínica de Síndrome Metabólico, Prediabetes y Diabetes de la Fundación Oftalmológica de Santander*). This analysis considered prevention of DM2 new cases and the cost-benefit of early and aggressive treatment of risk factors, such as abdominal obesity, nonalcoholic fatty liver disease, hypertension, and dyslipidemia, on prevention of DM2 complications. The IDPP results were used regarding the use of a single 500 mg/day dose of metformin and the necessity to treat of seven subjects with prediabetes to prevent one new case of diabetes. Thus, in Colombia, the cost of treating an IGT patient is US$ 826 annually, lower than the cost for treating DM2. The projected cost of metformin treatment for seven patients with prediabetes can avoid the cost of treating a non-complicated diabetic patient with metformin + glimepiride after 2.9 years; with metformin + DPP4 inhibitors after 2.6 years; and with metformin + insulin after 1.9 years. That is to say, after 2.3 years, the cost of primary prevention in a prediabetes patient has been recovered. In summary, the expert panel concluded that in Latin America, preventive treatment through lifestyle changes and metformin are cost-effective interventions.

**TABLE 2.** Estimated cost of treating impaired glucose tolerance to prevent a new case of type 2 diabetes versus cost of treating type 2 diabetes at different stages, 2013

Condition	Costs (2013 US$)			
	Clinical management	Drugs	Monthly cost	Total annual cost
Prediabetes	632	194	69	826
Type 2 Diabetes				
MTF^a^ + GMP^b^	1 304	651	163	1 955
MTF + DPP4^c^	1 304	887	183	2 191
MTF + INSUL^d^	1 304	1 726	253	3 030
Dialysis	18 813	1 726	1 712	20 539

a Metformin.

b Glimepiride.

c Sitagliptin.

d Glargine insulin.

Note: Marginal costs and out-of-the pocket expenses not included.

***Source:*** Prepared by the authors from the study data and data from the *Fundación Oftalmológica de Santander, Clínica de Prediabetes*, Bucaramanga, Colombia.

#### Limitations.

Since the information in this study was retrieved by a panel of experts and not through a systematic review, there may have been some omissions of scientific literature. However, a subsequent search of PubMed Central and LILACS databases on the use of diabetes risk scores in Latin America did not result in any additional publications for inclusion.

In addition, the panel comprised experts from the Latin American countries that have conducted the most research in the field of prediabetes, which may have led to over/under-representing findings from particular countries.

### RECOMMENDATIONS

The expert panel defined five key statements regarding prediabetes in Latin America:

1. Prediabetes is a clinical and public health problem in Latin America, underscored by its impact on cardiovascular disease and mortality. Due to a lack of prediabetes screening in Latin America, the current prevalence is unknown; however, there is reason to believe it ranges from 10% – 20%.
2. Health care systems in Latin America do not routinely perform prediabetes diagnostics and/or treatment and do not have strategies for doing so. Even though the region has well-trained health care professionals, there seem to be various obstacles, such as economical limitations, lack of standardized diagnostic methods (HbA1c, oral glucose tolerance test), and an absence of political willingness. Awareness need be raised among policymakers regarding the importance of timely screening and treatment of prediabetes. Actions should include algorithms to screen high-risk individuals using a two-step strategy (risk assessment tool followed by diagnostic invasive blood tests), plus pharmaceutical treatment and lifestyle changes.
3. Prediabetes risk detection tools include application of FINDRISC and implementation of fasting glucose assessment protocols, glucose tolerance test, and HbA1c. Screening tools, such as FINDRISC, efficiently identify people at high risk of abnormal glucose tolerance. These tools have been validated in some Latin American countries. We recommend the validation and implementation of the FINDRISC throughout the region to identify people at risk, using an algorithm for either fasting, 2-hour glucose, or HbA1c measures, or all three. These algorithms shall be part of national and regional clinical practice guidelines for the detection and prevention of type 2 diabetes in Latin America. The importance of prediabetes and its detection should be communicated on a large-scale at the population level by the Ministries of Health using public awareness campaigns, and also, by all health care providers screening their patient population for those at risk. National Diabetes or Endocrinology Associations can lead or facilitate communication among all stakeholders.
4. Important actions for prediabetes treatment include lifestyle changes, multidisciplinary education, and use of metformin as a therapeutic alternative. There is sufficient scientific evidence to conclude that type 2 diabetes can be prevented and/or delayed by interventions targeting lifestyle (especially physical activity and diet) and metformin. Although lifestyle interventions have had a larger effect than metformin, medication can provide an excellent option for certain patients. To implement lifestyle changes, both a population and a high-risk strategy are needed. The population strategy should be developed under the leadership of the Ministries of Health using the main concepts of health promotion, including the theory of diffusion of innovations. It should aim to decrease the prevalence of obesity and overweight in the population and to increase physical activity to at least 150 minutes/week. To succeed, this strategy must include the private and public sectors and interventions that target the entire life cycle. The high risk strategy, on the other hand, needs to be implemented within primary health care. It should offer individual and/or group advice on how to change lifestyle, and incorporate behavior change models, such as the Prochaska model. The primary health care sector must understand the importance and benefits of lifestyle interventions for those with prediabetes so that the burden of type 2 diabetes and its complications can be avoided.
5. It is important to support patient records and epidemiology studies in Latin America. Because most epidemiological studies on diabetes prevalence in Latin America rely on self-reported measurements, large population-based epidemiological studies using HbA1c and oral glucose tolerance tests are needed to estimate the real prevalence of IFG and IGT. Furthermore, patient records may provide important information on the number of people with prediabetes; however, these registries need to be validated and regularly updated to provide the necessary information on consultation, follow-up, and treatment of people with prediabetes.

## CONCLUSIONS

After reviewing the published evidence, the expert panel concluded that prediabetes in Latin America is an important public health problem, one that is unfortunately not well understood by the health sector. It is crucial that cost-effective preventive treatments, namely lifestyle changes and medications, such as metformin, be routinely offered and accessible. Moreover, it is necessary to improve prediabetes identification and management at the primary care level.

## Acknowledgements.

The authors thank Noel Barengo for his review of the manuscript and Paul Antony Camacho for permission to use data from the *Fundación Oftalmológica de Santander* to calculating the cost of treating prediabetes.

## Funding:

All of the authors received the financial support of Merck KGaA (Darmstadt, Germany) to participate in the meeting in Lima, Peru (travel and living expenses).

## Disclaimer.

Authors hold sole responsibility for the views expressed in the manuscript, which may not necessarily reflect the opinion or policy of the *RPSP/PAJPH* and/or PAHO.
